# Study of the link between hemotopoietic and skeletal systems in patients attending a referral center for osteoporosis

**DOI:** 10.1007/s40618-023-02095-3

**Published:** 2023-04-15

**Authors:** J. Pepe, L. Colangelo, V. De Martino, M. Occhiuto, D. Iervolino, P. Pasqualetti, S. Minisola, C. Cipriani

**Affiliations:** 1 Department of Clinical, Internal, Anesthesiological and Cardiovascular Sciences, “Sapienza” University of Rome, Viale del Policlinico 155, 00161 Rome, Italy; 2 Department of Public Health and Infectious Diseases, Section of Medical Statistics, Sapienza University of Rome, Rome, Italy

**Keywords:** Fracture, Osteoporosis, RDW, Hematocrit

## Abstract

**Purpose:**

To investigate the link between hematopoietic and skeletal tissues in patients with fragility fractures.

**Methods:**

We retrospectively analyzed the medical records of women older than 40 years who attended the Bone Disease Unit of “Sapienza” University of Rome for their first visit for osteoporosis from January 2020 to June 2022.

**Results:**

Fragility fractures were found in 61.8% of the sample. In particular, vertebral fractures in 35.5%, femoral fractures in 6.3%, Colles fractures in 16.5% and non-vertebral non-hip in 42.5%. Fractured patients were significantly older compared to non-fractured, had lower mean values of lumbar spine (*p* = 0.01), and femoral neck BMD (*p* = 0.007). A red blood cell distribution width (RDW) value higher than 15% was observed four times more in those with fractures compared to non-fractured patients (8.9% vs 2%, *p* = 0.01) and was associated with vertebral fracture after adjusting for age, BMI, menopause, nutritional status, smoking, osteoporosis and anemia (OR = 4.1, 95% CI 1.6–11.4, *p* = 0.003). Hematocrit was negatively associated with hip fracture also adjusting for age, BMI, menopause, nutritional status, smoking, osteoporosis (*p* = 0.025).

**Conclusion:**

Our study demonstrates that RDW values were significantly associated with vertebral fracture and hematocrit with hip fracture. Since both parameters are included in the initial evaluation of patients with suspected bone fragility, our results should push doctors to look at these values with no incremental cost for national health services.

## Introduction

Complete blood cell count is an integral part of the initial biochemical evaluation of osteoporotic patients. Recently, several investigators examined the possible association of red, white cells and platelets numbers with osteoporosis even though controversial results have been reported [1–15]. In particular, some studies have shown that anemia is a risk factor for both low Bone Mineral Density (BMD) and the risk of fracture, while others studies did not [1–7].

As a part of an automated complete blood count, red blood cell distribution width (RDW) represents the variation in size of the red blood cells, which is commonly used in the differential diagnosis of anemia. Recently, RDW is being increasingly recognized as a global marker of chronic inflammation and oxidative stress; elevated values in men have been associated with 2.8 times higher risk of hip fractures than men in the lowest group [8]. In a sample of both sexes, RDW was also associated with prevalent vertebral fractures [9].

Concerning white blood cells, an elevated peripheral blood neutrophil-to-lymphocyte ratio (NLR) levels has been reported in osteoporotic patients compared with osteopenic ones. However, also in this case, discordant results have been published [10, 11]. Fisher et al., reported that high NLR can be considered as a potential indicator of bone fracture [13]. This ratio can reflect the balance of the immune response and has been reported to be an independent and inexpensive predictor of multiple outcomes in many inflammatory and immune diseases. In addition, this study demonstrated that NLR has close relationship with *C*-reactive protein levels [13].

In some of the previous studies, the association between both osteoporosis/fractures and an alteration of red, white and platelet cell series, could possibly be related to proinflammatory cytokines alteration and oxidative stress [16–19]. Furthermore, one of the reasons underlying discordant data may possibly be due to inclusion of patients of both sexes and the correction, only in few studies, for malnutrition or anemia that may have biased the results.

The aim of our study was to investigate the association between peripheral blood cell counts (red and white blood cells, platelets) and fragility fractures adjusting for multiple confounding parameters such as nutritional status, osteoporosis, anemia and smoking.

## Methods

We retrospectively analyzed the medical charts of women older than 40 years who attended the Bone Disease Unit of “Sapienza” University of Rome for their first visit for osteoporosis from January 2020 to June 2022. Exclusion criteria for participation in the study were: Body Mass Index (BMI) > 30 kg/m^2^, creatinine clearance < 60 ml/min, neoplasias, active inflammatory diseases, known secondary causes of osteoporosis, intake of drugs that affect bone or iron metabolism, with the exception of vitamin D supplementation, anticoagulant and antiaggregant. Each subject underwent bone mineral density (BMD) measurement at the lumbar spine (L1–L4) and hip (neck and total hip) using a HOLOGIC QDR 4500 [20]. Subjects also had a standard spine X-ray to evaluate vertebral deformity according to Genant’s method. Fractured lumbar vertebrae were excluded from BMD measurement. Osteoporosis was diagnosed considering *T* score ≤ − 2.5 SD at the lumbar or femoral site by DXA scan [21]. We considered as osteoporotic fracture every atraumatic or low-energy trauma clinical fracture (with the exclusion of fractures of the fingers, toes, face and skull) and morphometric vertebral fracture.

Complete blood count parameters were analyzed with an automated hematology analyzer at our laboratory in 92% of cases, the remaining being measured outside our hospital. The reference range of RDW, in our laboratory, is 11–15%. Main biochemical parameters such as total protein, albumin, calcium, phosphate and creatinine were also measured as previously described [22]. The study was approved by the local Ethics Committee of University.

### Statistical analysis

To test the normality of the data, we used the Shapiro–Wilk test. Whenever an adequate fit to Gaussianity (checked also by visual inspection of box-plots and probability-plots) was nor observed neither achievable by means of data transformation, ordinal statistics and corresponding non-parametric tests were used. In the other cases, parametric statistics and tests were applied.

When the analysis concerned more than two groups, ANOVA or Kruskal–Wallis procedures were used, followed by Bonferroni adjusted post-hoc *t* test or Mann–Whitney or binomial tests for pairwise comparisons.

Logistic models to test association between fractures and blood counts were carried out, adjusting for the following parameters: age, BMI, menopause, nutritional status (assessed by means of total protein, albumin, calcium, phosphate values), smoking, osteoporosis and anemia, defined in women as a Hb levels < 12 g/dL according to the WHO threshold [23]. The statistical analyses were carried out with R software, version 4.1.1 (2021-08-10). The results were considered significant when the probability value was less than 5% (*p* value < 0.05).

## Results

We enrolled 400 women, median age 69.00 [61.00, 76.00] years old, 3.7% of them being premenopausal. Mean age at menopause was 48.6 ± 4.9 years, BMI was in the normal range with a median of 23.2 kg/m^2^ (IQR: 21.05, 25.71). Anemia was found in 30 women (7.5%; Wilson 95% CI 5.3–10.5%).

After initial DXA scan, according to lumbar and/or femoral T-score values, 33.5% (95% CI 29.1–38.3%) of the sample was diagnosed as having osteopenia, 63% (95% CI 58.2–67.6%) osteoporosis and 3.7% (95% CI 2.1–5.8%) had T-score values in the normal range. There were no differences between these three groups concerning anthropometric parameters, except for a mean lower BMI in osteoporotic patients (23 ± 3.7 kg/m^2^) compared to both osteopenic (24.7 ± 4 kg/m^2^) and normal subjects (26.3 ± 4 kg/m^2^), *p* < 0.001. The osteoporotic patients were more frequently smokers (23.1%), compared to both osteopenic (11.3%) and normal subjects (13.3%, *p* = 0.016). Mean values of other biochemical parameters were not significantly different in the three groups (Table 1).

**Table 1:** Anthropometric, biochemical and bone mineral density values in patients subdivided according to BMD. Results are presented as median and [interquartile range] and mean

	Normal subjects	Osteopenic patients	Osteoporotic patients
Number of patients	15	134	251
Age (years)	64.2 (10.7)	67.4 (11.4)	69.1 (10.0)
Menopause (%)	14 (93.3)	125 (93.3)	247 (98.0)
Years since menopause	11.0 [4.75, 26.0]	21.0 [13.0, 29.0]	20.0 [13.0, 28.0]
BMI (kg/m^2^)	26.3 (4.1)	24.7 (4.0)	23.0 (3.7)*^§^
Smoking (%)	2 (13.3)	15 (11.3)	58 (23.1)^§^*
L1–L4 BMD (g/cm^2^)	1.12 (0.23)	0.90 (0.13)*	0.75 (0.12)*^§^
L1–L4 T-score (SD)	0.83 (1.31)	− 1.42 (1.06)*	− 2.85 (1.00)*^§^
Femoral neck BMD (g/cm^2^)	0.86 (0.12)	0.70 (0.08)*	0.60 (0.10)*^§^
Femoral neck T-score (SD)	− 0.12 (0.81)	− 1.63 (0.58)*^§^	− 2.54 (0.73)*^§^
Femoral total BMD (g/cm^2^)	0.95 (0.11)	0.79 (0.10)*	0.70 (0.09)*^§^
Femoral total T-score (SD)	− 0.17 (0.90)	− 1.34 (0.77)*	− 2.11 (0.81)*^§^
Red blood cells (× 10^6^ microL)	4.684 (4.13)	4.610 (4.48)	4.568 (5.23)
Hemoglobin (g/dL)	13.65 (1.21)	13.49 (0.88)	13.48 (1.17)
Hematocrit (%)	41.34 (2.77)	41.15 (3.30)	40.74 (5.02)
RDW > 15 (%)	1 (6.7)	8 (6.0)	16 (6.3)
Platelets (× 10^3^ microL)	248.9 (5.99)	244.7 (5.95)	247.6 (6.09)
White cells (× 10^3^ microL)	6.33 (1.32)	6.15 (1.68)	6.18 (2.13)
Creatinine (mg/dL)	0.80 [0.71, 0.84]	0.77 [0.66, 0.88]	0.74 [0.66, 0.84]
Calcium (mg/dL)	9.60 [9.30, 9.90]	9.60 [9.30, 9.90]	9.50 [9.20, 9.80]
Phosphate (mg/dL)	3.70 [3.30, 3.90]	3.80 [3.40, 4.20]	3.72 [3.40, 4.00]
Total protein (g/L)	6.90 [6.75, 7.35]	7.10 [6.80, 7.40]	7.04 [6.70, 7.30]
Albumin (g/L)	4.00 [3.77, 4.26]	4.06 [3.78, 4.30]	4.06 [3.78, 4.30]
Neutrophil (× 10^3^ microL)	3.57 [3.05, 4.01]	3.25 [2.67, 4.00]	3.25 [2.71, 3.99]
Lymphocyte (× 10^3^ microL)	1.92 [1.70, 2.72]	1.89 [1.59, 2.34]	1.89 [1.55, 2.25]
Monocyte (× 10^3^ microL)	0.41 [0.32, 0.48]	0.42 [0.33, 0.51]	0.43 [0.34, 0.52]
Eosinophil (× 10^3^ microL)	0.13 [0.11, 0.24]	0.13 [0.09, 0.21]	0.13 [0.09, 0.20]
Basophil (× 10^3^ microL)	0.05 [0.03, 0.05]	0.03 [0.02, 0.05]	0.04 [0.02, 0.05]
NLR	1.91 [1.40, 2.18]	1.76 [1.29, 2.17]	1.64 [1.36, 2.18]
MLR	0.20 [0.15, 0.23]	0.22 [0.18, 0.27]	0.22 [0.18, 0.28]

*MLR* monocyte-to-lymphocyte ratio, *NLR* neutrophil-to-lymphocyte ratio, *RDW* red blood cell distribution width, *BMD* Bone Mineral Density *L*_*1*_*–L*_*4*_ lumbar spine

*Bonferroni adjusted *p* < 0.05 vs. Normal

^§^Bonferroni adjusted *p* < 0.05 vs. Osteopenic

Fragility fractures were found in 61.8% (95% CI 56.9–66.4%) of the sample and, in particular, vertebral fractures in 35.5% (95% CI 31.0–40.3%), femoral fractures in 6.3% (95% CI 4.3–9.1%), Colles fractures in 16.5% (95% CI 13.2–20.5%) and non-vertebral non-hip fractures (peripheral fractures) in 42.5% (95% CI 37.8–47.4%).

Patients with fractures were significantly older (70.35 ± 9.91) compared to those without (65.06 ± 10.76 *p* < 0.001); they had significantly mean lower lumbar spine BMD (*p* = 0.01), and femoral neck BMD (*p* = 0.007) compared with those without fractures, as shown in Table 2. There were no differences in main comorbidities or medication taken in the two groups (Table 3).

**Table 2:** Anthropometric, biochemical and bone mineral density values in patients subdivided according to fragility fractures. Results are presented as median [interquartile range] and mean

	Patients without fractures	Patients with fractures
Number of patients	153	247
Age (years)	65.06 (10.76)	70.35 (9.91)*
Menopause (%)	145 (94.8)	240 ( 97.2)
Years since menopause	15.00 [9.00, 24.00]	23.00 [15.00, 30.00]*
BMI (kg/m^2^)	23.42 (3.90)	23.90 (3.92)
Smoking (%)	25 (16.3)	50 (20.4)
L1–L4 BMD (g/cm^2^)	0.84 (0.14)	0.80 (0.16)**
L1–L4 T-score (SD)	− 2.08 (1.33)	− 2.32 (1.39)
Femoral neck BMD (g/cm^2^)	0.66 (0.12)	0.63 (0.11)°
Femoral neck T-score (SD)	− 2.04 (0.90)	− 2.20 (0.89)
Femoral total BMD (g/cm^2^)	0.75 (0.12)	0.74 (0.11)
Femoral total T-score (SD)	− 1.74 (0.99)	− 1.80 (0.89)
Red blood cells (× 10^6^ microL)	4.562 (5.28)	4.604 (4.74)
Hemoglobin (g/dL)	13.53 (1.07)	13.47 (1.09)
Hematocrit (%)	41.01 (4.63)	40.85 (4.32)
RDW > 15 (%)	3 (2.0)	22 (8.9)**
Platelets (× 10^3^ microL)	248.6 (5.79)	245.6 (6.19)
White cells (× 10^3^ microL)	6.12 (1.59)	6.21 (2.16)
Creatinine (mg/dL)	0.76 [0.65, 0.87]	0.74 [0.67, 0.85]
Calcium (mg/dL)	9.60 [9.27, 9.80]	9.60 [9.20, 9.89]
Phosphate (mg/dL)	3.80 [3.40, 4.10]	3.80 [3.40, 4.03]
Total protein (g/L)	7.10 [6.70, 7.30]	7.07 [6.70, 7.37]
Albumin (g/L)	4.06 [3.81, 4.29]	4.06 [3.78, 4.31]
Neutrophil (× 10^3^ microL)	3.32 [2.75, 3.99]	3.21 [2.67, 4.00]
Lymphocyte (× 10^3^ microL)	1.86 [1.58, 2.30]	1.91 [1.55, 2.25]
Monocyte (× 10^3^ microL)	0.42 [0.33, 0.51]	0.43 [0.34, 0.52]
Eosinophil (× 10^3^ microL)	0.13 [0.09, 0.20]	0.14 [0.09, 0.21]
Basophil (× 10^3^ microL)	0.04 [0.02, 0.05]	0.03 [0.02, 0.05]
NLR	1.69 [1.37, 2.16]	1.66 [1.33, 2.28]
MLR	0.22 [0.18, 0.27]	0.22 [0.18, 0.28]

*MLR* monocyte-to-lymphocyte ratio, *NLR* neutrophil-to-lymphocyte ratio, *RDW* red blood cell distribution width, *BMD* Bone Mineral Density, *L*_*1*_*–L*_*4*_ lumbar spine

*p < 0.001

°*p* = 0.007

**p = 0.01

**Table 3:** Comorbidities and medication in patients according to fragility fractures

	Patients without fractures	Patients with fractures
Number of patients	153	247
Vitamin D supplements (%)	125 (81.7)	208 (84.2)
Calcium supplements	45 (29.4)	75 (30.4)
Diabetes (%)	5 (3.3)	16 (6.5)
Hypertension (%)	40 (26)	70 (28)
Thyroid nodules (%)	47 (30.7)	80 (32.4)
Dyslipidemia (%)	34 (22.4)	69 (27.9)

Concerning biochemical parameters, the only significant difference was a higher number of patients with RDW > 15% in fractured patients compared to non-fractured ones, as shown in Table 2. A RDW value higher than 15% was observed four times more in fractured patients compared to those without (8.9% vs 2%, *p* = 0.01). Considering the diabetic population only 3 subjects had a RDW value higher than 15%, which is not a statistically significant difference when compared to the number of those with RDW > 15% (n = 22) in the population without diabetes (*p* > 0.05).

Furthermore, in the all population, a RDW value higher than the upper normal value (i.e., > 15%) was associated with vertebral fractures (OR = 4.05, 95% CI 1.8–11, *p* = 0.001). This level of association remained stable when potential confounders were taken into account as shown in Table 4. In particular, high RDW value remains associated with vertebral fractures even after adjusting for all considered possible confounders: age, BMI, menopause, nutritional status (total protein, albumin, calcium phosphate), smoking, osteoporosis and anemia (OR = 4.1, 95% CI 1.67–11.4, *p* = 0.003), (Table 4).

**Table 4:** The association between RDW and fractures (binary dependent variable) after adjusting for single and multiple potential confounders

Models	OR for RDW > 15% vs. ≤ 15% (95% CI)	*p* value
No adjustment	4.05 (1.8/11.0)	0.001
Adjusted by age	4.05 (1.6/11.0)	0.003
Adjusted by osteoporosis	4.05 (1.8/11.0)	0.001
Adjusted by anemia	4.05 (1.7/10.2)	0.001
Adjusted by age, BMI, menopause	4.05 (1.6/11.0)	0.003
Adjusted by age, BMI, menopause, smoking, osteoporosis	4.05 (1.6/11.0)	0.003
Adjusted by age, BMI, menopause, smoking, osteoporosis, nutritional status (total protein, albumin, calcium, phosphate)	4.05 (1.7/11.0)	0.003
Adjusted by age, BMI, menopause, smoking, osteoporosis, nutritional status (total protein, albumin, calcium, phosphate), anemia	4.1 (1.6/11.4)	0.003

Considering other blood parameters, monocyte-to-lymphocyte ratio (MLR) was associated with vertebral fractures (Beta = 2.4, 95% CI 0.24–4.7, *p* = 0.031), also after adjustment for osteoporosis (Beta = 2.4, 95% CI 0.24–4.7, *p* = 0.037); however, it was not significantly associated after adjustment for age, menopause, BMI and nutritional status. A low hematocrit was associated with hip fractures (OR for 1-unit increase = 0.90, 95% CI 0.82 -0.99, *p* = 0.025), also after adjustment for age, BMI, menopause, nutritional status, smoking, osteoporosis (Table 5). Considering drugs that may interfere with hematocrit, we found no differences in the number of patients taking diuretics between fractured hypertensive vs non-fractured hypertensive patients (20/40 vs 32/70, *p* = 0.69). Among the diabetic population, a small number of patients were taking SGLTi therapy, without any difference in the number treated in the diabetic fractured group vs the diabetic non-fractured group (2/5 vs 4/16, *p* = 0.5).

**Table 5:** The association between hematocrit value with hip fractures after adjusting for single and multiple potential confounders

Models	OR for 1-unit increase of hematocrit (95% CI)	*p* value
No adjustment	0.90 (0.83/0.96)	0.0046
Adjusted by age	0.92 (0.84/0.98)	0.014
Adjusted by osteoporosis	0.91 (0.84/0.97)	0.0075
Adjusted by age, BMI, menopause	0.92 (0,84/0.98)	0.015
Adjusted by age, BMI, menopause, smoking, osteoporosis	0.93 (0.85/0.99)	0.033
Adjusted by age, BMI, menopause, smoking, osteoporosis, total protein, albumin, calcium, phosphate	0.90 (0.82/0.99)	0.025

We found no association between platelets number and fragility fractures considered as a whole.

## Discussion

A number of “in vitro” and “in vivo” studies have consistently shown a two-way influence between skeletal cells and hematopoietic system [24]. RDW, a parameter related to erythropoiesis that measures variation in erythrocytes’ size, is considered a marker of biological aging; therefore, it is straightforward its evaluation in osteoporosis, a typical disease of aging population.

We showed an association between higher RDW values and vertebral fractures after adjusting for multiple potential confounding variables including smoking which has never been considered in this setting. It is important to consider cigarette smoking, because this life style habit is recognized as an independent risk factor for the development of osteoporosis, and it is linked to an enhanced oxidative stress [25]. Our results are in line with prior investigations that have found an association between higher RDW values and fractures, although not adjusting the data for multiple confounding parameters including smoke [8, 9]. For example, Kim et al. [8] prospectively followed 3635 men for an average period of 8.1 years. They found that the risk of hip as well as all clinical fractures increased along with higher RDW values. The discrepancy with our study showing only an increased risk with spine fracture can be easily explained by a number of factors. Gender, study design, different fracture event in the relatively small population we studied are the most important.

One of the possible hypotheses linking RDW to fractures may be chronic inflammation. In a recent study, RDW independently predicted in-hospital mortality, 90-day mortality, and hospital and ICU length of stay in fractured patients admitted to ICU, highlighting the link between RDW and inflammation leading to a worst outcome [26]. Indeed, inflammation suppresses bone marrow erythropoiesis and consequently affects the heterogeneity of RBC size [27] while it may also be responsible for the development of fragility fractures. Also, clonal hematopoiesis has been suggested as a causative factor. Clonal hematopoiesis is a somatic mutation that occurs in hematopoietic stem cells with advancing age and is known to be related with several aging-related diseases [28]. As a consequence, the disturbed hematopoiesis that occurs with clonal hematopoiesis causes both a variation in RBC size and a negative impact on skeletal resistance [29]. Intriguingly, we found such a correlation only with vertebral fractures that is at a skeletal site where bone marrow is active throughout life.

We also firstly detected an association between MLR and vertebral fracture. Peripheral blood monocytes can further differentiate into many kinds of cells such as macrophages, dendritic cells and osteoclast cells. MLR could be considered another marker of inflammation linked to vertebral fracture. However, after adjustment for age, BMI and menopause, this association became no longer significant.

Our data showed that hematocrit is negatively associated with hip fracture. Although, the exact mechanism by which hematocrit is associated with fracture is still unclear, animal models have shown that osteoblast ablation leads to deficiency in the progenitors of multiple hematopoietic cell lines [30]. Interestingly, osteoblasts may directly modulate red blood cell production potentially through production of erythropoietin [31, 32]. RDW may be more linked to inflammation and vertebral fracture, while anemia may affect muscle supply thus increasing the risk of falls and hip fracture. Another hypothesis might be that anemia also results from iron deficiency and/or vitamin B12 and folic acid, these deficiencies may induce peripheric polyneuropathy which increases the risk of falls. However, we did not measure these micronutrients and this could be considered a limit of our study.

Of note, we only included low-energy trauma fractures, while a large population-based cohort in the South Korea, which showed an association of fracture with anemia included all types of fractures, including high energy trauma [33]. We did not find any association between platelets and fragility fracture, in line with previous studies showing no consistent associations between platelet count and bone mineral density (BMD) [4].

Our study has some limits. First, this was a single center study, not including men. Second, in our sample, we did not rule out subclinical hypothyroidism, which might influence RDW value. Indeed, RDW has been recently considered a marker of subclinical hypothyroidism [34]. Third, we are not able to elucidate a clear pathogenetic mechanism linking RDW with vertebral fracture and hematocrit with hip fractures even though the theoretical mechanisms proposed giving a differential effect are intriguing. Moreover, a recent study, in a large cohort of community-dwelling older adults, shows how elevated RDW is independently associated with a higher frailty risk [35].

In conclusion, our study demonstrated that RDW values was significantly associated with vertebral fracture and hematocrit with hip fracture. Since both parameters are included in the initial evaluation of patients with suspected bone fragility, our results should push doctors to look at these values with no incremental cost for national health services.

## Data Availability

The datasets generated during and/or analyzed during the current study are available from the corresponding author on reasonable request.
